# Perilymphatic fistula in guinea pigs: natural evolution versus surgical treatment

**DOI:** 10.1590/S1808-86942010000200006

**Published:** 2015-10-19

**Authors:** Ektor Tsuneo Onishi, Yotaka Fukuda

**Affiliations:** PhD in Medicine - Federal University of São Paulo - Escola Paulista de Medicina. Coordinator of the Tinnitus Ward - Department of Otorhinolaryngology and Head and Neck Surgery -UNIFESP-EPM; Associate Professor - Federal University of São Paulo - Escola Paulista de Medicina, Associate Professor - Otology and Neurotology Program - UNIFESP-EPM

**Keywords:** fistula, round window, perilymph

## Abstract

Perilymphatic fistulas still represent a major treatment challenge. In some cases, its surgical closure can reduce auditory and vestibular sequelae.

**Aim:**

to compare the behavior of cochlear window perilymphatic fistulas in guinea pigs as to their natural evolution and immediate surgical closure.

**Materials and Methods:**

Experimental study. Forty guinea pigs were submitted to cochlear window membrane lesion and randomly broken down into two groups: open fistula (OF) and surgically closed fistula (SCF). We found the summation potential (SP) and action potential (AP) latencies and amplitudes and the SP/AP ratio at three times: pre-fistula (PRE), immediate post-fistula (IPF) and late post-fistula (LPF).

**Results:**

There was a significant drop in amplitudes and raise in SP and AP latencies among the times studied. As to the SP/AP ratios, there was a reduction between PRE and IPF, both were significant. There was no behavior difference between the OF and SCF.

**Conclusions:**

Within the time frame considered, guinea pigs submitted to cochlear window membrane lesions evolved with a worsening in potentials and latencies. Despite the partial improvement in electrophysiological parameters, surgical closure did not prove statistically more effective than natural evolution.

## INTRODUCTION

Considered as an abnormal shunt between the cochlea and the middle ear cavity, the perilymphatic fistula (PF) can be the result of chronic otitis media complications, head injury, acoustic trauma and barotrauma, amongst others, and we must stress the fistulas caused by congenital malformations responsible for repetition meningitis spells, the iatrogenic ones and the ones related to ear surgery, especial stapes surgeries^1^, ^2^, ^3^, ^4^, ^5^.

There are reports of a great clinical expression variability, which makes it difficult, not only the diagnosis, but it also impairs the attempts to classify or standardize its signs and symptoms. It is more common to find reports of sensorineural dysacousia of varied degrees of sudden or fluctuating onset, associated with vertigo or dizziness and tinnitus. Conductive hearing loss may also occur, in a lower frequency^1^^,^^4^^,^^6^.

Many are the treatments proposed, as are the results achieved^7^^,^^8^. If on the one hand there is a major concern regarding the cost generated by the surgical procedure and hospital stay, medium and long term sequelae of the PF persistence and its consequences - the need for rehabilitation and refitting of the individual who suffers from hearing loss and vertigo - must also not be forgotten, since they directly impact the patient's quality of life. There is evidence that normalizing the perilymphatic pressure is needed in order to recover the membranous labyrinth and bring about early improvement to the vestibular systems^9^, ^10^, ^11^, ^12^. Such fact can not simply be ignored, since it is based on a viable treatment option that must be made available for the patients, especially when considering the poor results obtained from the treatment of sensorineural hearing loss in general.

The search for a more precise diagnosis led some authors to study hearing through experimental perilymphatic fistulas and its evolution by means of electrophysiological tests^13^, ^14^, ^15^, especially electrocochleography with different methodologies and degrees of recovery^16^, ^17^, ^18^.

The great diversity of approaches and their results^19^^,^^20^, as well as the need to obtain more objective criteria in order to choose the type of treatment to be instated in these cases (surgical or clinical-expecting), motivated us to develop this study which aims at comparing the results of round window perilymphatic fistulas kept open with the ones surgically closed by fat tissue, using electrocochleography.

## MATERIALS AND METHODS

Experimental study. The project was fully approved by the Ethics in Research Committee of the Institution (protocol # 557/03). We selected forty adult male guinea pigs (300 to 500 grams). The animals were submitted to anesthesia by ketamine chlorate (70mg/kg in weight) and xylazine chlorate (6mg/kg of weight) intraperitoneally. We did a retroauricular approach through a skin incision in the muscle and cellular subcutaneous tissue up to complete exposure of the mastoid bulla which was opened with a high rotation burr until it was possible to identify the round window niche under the surgical light microscope. The animal was taken to an acoustically treated room in order to be submitted to an electrocochleography (0.1 ms click at 90 dB HL, 100 Hz high pass filter, 3 KHz low pass). The stimulus happened through TDH 39 phones positioned at a fixed distance of 2 centimeters from the external acoustic meatus, and each register was duplicated at least twice to check for its accuracy. After obtaining the electrocochleography traces (pre-fistula exam - PRE), they were submitted to a crossed incision on the round window membrane with the help of a one millimeter micro-hook under microscopic view, trying not to damage the membranous structures of the inner ear. It was possible to observe the perilymph oozing through the fistula, which was not removed by aspiration. Immediately after making the fistula, we carried out another electrocochleography exam (immediate post-fistula exam - IPF) and the ears were randomly assigned to two study groups: fistula maintenance (open fistula group - OF) or its immediate closure (Closed Fistula Group - CF) with fat tissue, which fragments were obtained through the same skin incision and carefully placed on the round window. The skin was sutured in one single plane with non-absorbable wire. After having recovered from the anesthesia, the animals were kept in their cages with full access to food and water, under controlled temperature and proper technical care. After two weeks, they were once again anesthetized and approached through the retroauricular pathway in order to check the cavity and rule out the presence of otitis media, and a new electrocochleography test was carried out (late post-fistula exam - LPF) in order to compare the results. We analyzed the following parameters: Summation Potential (SP) and Action Potential (AP) amplitudes and latencies and the relationship between these Summation Potential and Action Potential amplitudes (SP/AP ratio). The results were statistically analyzed through the Variance Analysis Test (ANOVA), with a 5% significance level.

## RESULTS

Of the forty guinea pigs with which we started the study, 10 (ten) were taken off because they died in the immediate post-op (because of anesthetic complications), or for having developed acute otitis media or secretory otitis media seen during the inspection of the mastoid bulla at the time of the third electrocochleography (PFT).

### Summation Potential Amplitude (SP)

**Graph 1:**
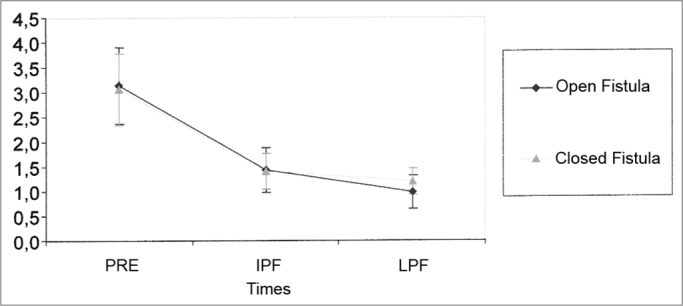
Mean (mV) and standard deviation (mV) values of the SP amplitude according to the different times (N=21) (Confidence interval for the mean: mean ± 1.96 * standard deviation / √ (n-1)) - Pre - IPF -immediate post-fistula LPF - late post-fistula.

Of the thirty guinea pigs, 9 were taken off this calculation because they did not have numerically accounting data (no response). Table 1 shows the values obtained for the mean, standard deviation and sample size studied in the groups at the different times of the experiment. We have noticed that there is a similarity between the mean values obtained in the PRE and PFI for both groups, with a small difference for the values at a third moment (PFT), greater in the CF group. The results from the application of the Variance Analysis Test (ANOVA) with a 5% significance level are shown on Table 2. There was a significant difference between the times, but not between the groups. Through multiple comparisons, we have noticed that in both groups there is a SP amplitude reduction, being more important between PRE and PFI. Although the CF Group presented a mild trend to PFT recovery, there was no statistically significant difference between the situations (Graph 1).

**Table 1:** Mean values (mV) and standard deviations (mV) of the SP amplitude at different times in the study.

Situation		PRE	IPF	Statistics
	Mean	3,14	1,43	0,97
Open Fistula Group (OF)	Standard deviation	1,74	1,03	0,78
	Size	21	21	21
	Mean	3,07	1,40	1,18
Closed Fistula Group (CF)	Standard deviation	1,65	0,82	0,61
	Size	21	21	21

**Table 2:** Results from the Variance Analysis Test (ANOVA) for SP amplitude.

Effect	F Significance (p)	Result
Situation	0.8823	Equal
Times	<0.0001 *	Different
Situation × Times	0.4828	Equal

### Action Potential Amplitude (AP)

**Graph 2:**
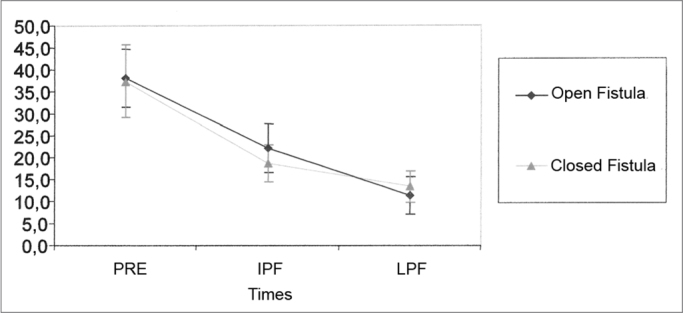
Mean (mV) and standard deviation (mV) values of the AP amplitude from the groups at different times (N=23) (Confidence interval for the mean: mean ± 1.96 * standard deviation / √ (n-1)) - PRE - pre-fistula IPF - immediate post-fistula LPF - late post-fistula.

Seven guinea pigs were taken off the study because they did not show numerically accountable data for this parameter. Table 3 shows the values obtained for the mean, standard deviation and sample size for the sample studied at the different times of the experiment. The results from the Variance Analysis Test (ANOVA) with a 5% significance level are shown on Table 4. Similarly to what happened with the SP amplitude, there was a significant difference between the times, but not between the groups. Through multiple comparisons, we've noticed that in both groups there was an important AP amplitude reduction between PRE and PFI and a lower reduction between PFI and PFT, without statistically significant difference between the situations (Graph 2).

**Table 3:** Mean values (mV) and standard deviation values (mV) of AP amplitude at the different times of the study.

Situation	Statistics	Pre	IPF	LPF
	Mean	38,13	22,18	11,35
Open Fistula Group (OF)	Standard deviation	15,77	13,20	10,09
	Size	23	23	23
	Mean	37,47	18,68	13,32
Closed Fistula Group (CF)	Standard deviation	19,70	10,10	8,48
	Size	23	23	23

**Table 4:** Variance Analysis Test (ANOVA) for AP amplitude.

Effect	F Significance (p)	Result
Situation	0.7754	Equal
Times	<0.0001 *	Different
Situation × Moments	0.3065	Equal

### Relationship between SP and AP (SP/AP ratio)

**Graph 3:**
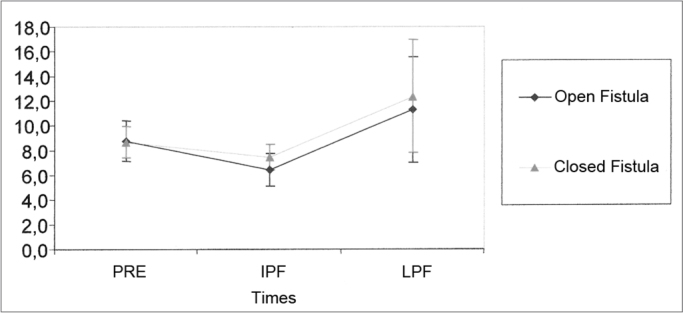
Mean (%) and standard deviation (%) values from the SP/AP ratio from the groups at different times (N=21) (confidence interval for the mean: mean ± 1.96 * standard deviation / √ (n-1)) - PRE - Pre-fistula; IPF - immediate post-fistula; LPF - late post-fistula.

We used 21 guinea pigs for this analysis. Table 5 shows the values obtained for the mean, standard deviation and sample size evaluated from the groups at the different times of the experiment. The results from the Variance Analysis Test (ANOVA) with a 5% significance level are shown on Table 6. There was a statistically significant difference between these times, but not between the groups. Through multiple comparisons, we noticed that in both groups the PRE and PFI and there was an increase in the SP/AP ratios between PFT and LPF, without statistically significant difference between the groups (Graph 3).

**Table 5:** Mean values (%) and standard deviation values (%) of the AP/SP ratio at the different times of the study.

Situation	Statistics	Pre	IPF	LPF
	Mean	8,76	6,34	11,24
Open fistula	Standard deviation	3,80	3,04	9,78
	Size	21	21	21
	Mean	8,66	7,38	12,36
Closed fistula	Standard deviation	2,90	2,35	10,52
	Size	21	21	21

**Table 6:** Results from the Variance Analysis Test (ANOVA) for the SP/AP ratio

Effect	F significance (p)	Result
Situation	0.4759	Equal
Times	0.0169 *	Different
Situation × Times	0.8518	Equal

### SP latency

**Graph 4:**
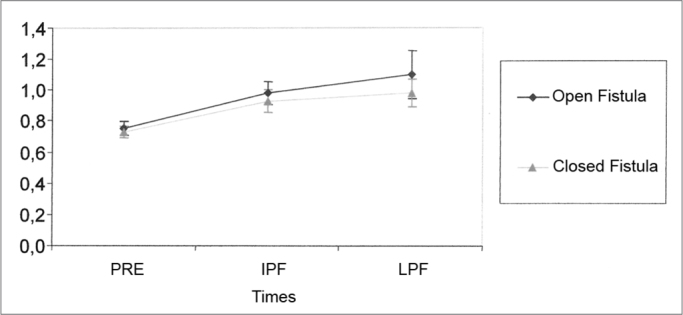
Mean (ms) and standard deviation (ms) values of the SP latency from the groups at different times (confidence interval for the mean: mean ± 1.96 * standard deviation / √ (n-1)) — PRE - Pre-fistula; IPF - immediate post-fistula; LPF - late post-fistula.

Nine guinea pigs were taken off the calculations of this parameter because they did not have numerically accountable data. Table 7 shows the values obtained for the mean, standard deviation and sample size studied in the group at the different times of the experiment. The results from the Variance Analysis Test (ANOVA) with a 5% significance level are shown on Table 8. There was a significant difference between the time periods, but not between the groups. By means of multiple comparisons we noticed that in both groups there was an increase in SP latency between PRE and PFT and between PFT and LPF. Although not significant, we noticed a trend towards a differentiated behavior between the situations in the PFT (Graph 4).

**Table 7:** Mean values (ms) and standard deviation values (ms) of the SP latency at the different times of the study.

Situation	Statistics	Pre	IPF	LPF
	Mean	0,75	0,98	1,10
Open Fistula Group (OF)	Standard deviation	0,10	0,16	0,35
	Size	21	21	21
	Mean	0,73	0,93	0,98
Closed Fistula Group (CF)	Standard deviation	0,08	0,17	0,20
	Size	21	21	21

**Table 8:** Results from the Variance Analysis Test (ANOVA) for the SP latency

Effect	F significance (p)	Results
Situation	0.1133	Equal
Times	<0.0001 *	Different
Situation × Times	0.4314	Equal

### AP latency

**Graph 5:**
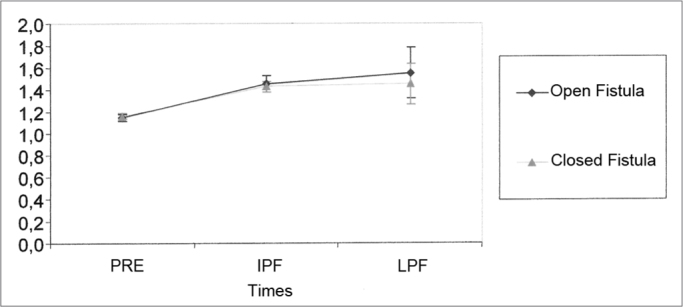
Mean (ms) and standard deviation (ms) values of the AP latency from the groups at different times (confidence interval for the mean: mean ± 1.96 * standard deviation / √ (n-1)) — PRE - Pre-fistula; IPF - immediate post-fistula; LPF - late post-fistula.

Of the thirty guinea pigs, 7 were taken off the calculations of this parameter for not presenting numerically accountable data. Table 9 shows the values obtained for the mean, standard deviation and sample size studied in the groups at the different times of the experiment. The results of the Variance Analysis Test (ANOVA) with a 5% significance level are presented on Table 10. There was a significant difference between the times, but not between the groups. Through multiple comparisons, we noticed that in both groups there was an AP latency increase between the PRE and PFT and the results were similar between the PFT and the LPF. Although not significant, there was a trend towards a differentiated behavior between the LPF situations (Graph 5).

**Table 9:** Mean values (ms) and standard deviation values (ms) of AP latency at the different times of the study.

Situation	Statistics	Pre	IPF	LPF
	Mean	1,15	1,45	1,55
Open Fistula Group (OF)	Standard deviation	0,07	0,18	0,56
	Size	23	23	23
	Mean	1,16	1,43	1,45
Closed Fistula Group (CF)	Standard deviation	0,06	0,11	0,44
	Size	23	23	23

**Table 10:** Results from the Variance Analysis Test (ANOVA) for AP latency

Effect	F significance (p)	Result
Situation	0.5667	Equal
Times	<0.0001 *	Different
Situation × Times	0.6116	Equal

## DISCUSSION

Let's consider the labyrinth as a system made up of ducts and canals filled by endolymph and perilymph, outlined by delicate membranes. The close contact with the adjacent structures makes it prone to suffer direct influence of hydrodynamic forces of the venous and arterial system and of the CSF in the subarachnoid space. Based on this principle, Goodhill^6^ proposed the existence of two possible routes or mechanisms which would cause membrane rupture. The explosive route by increase in CSF pressure transmitted through the cochlear aqueduct or internal acoustic meatus and the explosive pathway, where the increase in hydrostatic pressure in the middle ear would happen through the Eustachian Tube. Studies and experiments which followed were able to prove this theory^2^^,^^13^.

Considering the type and level of damage to the round window membrane, the review of experimental studies which have been held so far shows that the authors did basically a simple perforation or small lacerations on it^1^^,^^2^^,^^9^^,^^10^^,^^14^^,^^15^. For all the cases there was a trend towards lesion healing after a variety of mechanical traumas, even in the presence of otitis media, secretory otitis media or hemorrage^2^ within a mean time of two weeks, with recovery of electrophysiological parameters. It would not have been useful in our experiment to reproduce lesions of a similar degree; in other words, to compare open or closed fistulas, having this natural and spontaneous healing trend in guinea pigs. Therefore, we chose to create larger lesions on the round window, hoping to keep them open for a longer period which would be enough to analyze and compare the results.

The lesion of the round window membrane only does not cause significant hearing alterations^9^^,^^11^^,^^13^^,^^16^. As it happens in otospongiosis surgeries, when we cause an opening and exposure of the vestibule structures and we create a true perilymphatic fistula, without necessarily causing cochlear lesion. How can we explain the hearing loss associated with breaking the round or oval window membrane integrity, surgically or experimentally caused, when even in cases in which there is mild perilymph overflowing, dysacousia is not the inescapable result.

Simmons^3^ already had these doubts when he postulated the existence of a second lesion in some site of the membranous labyrinth, more specifically on the Reissner membrane - which would occur before, after or together with the first on the oval or round window. This small break on the Reissner membrane would allow the mixture of endo and perilymph, covering a larger extension and affecting areas more distant from the initial site, anatomically perfect, but with a functional loss caused by the change in cellular electrical potentials. The distance of this lesion from the basal cochlear region would be a determining factor to configure the hearing loss, and also a prognostic factor as to the level of recovery, depending if its healing would happen before, after or together with the first, or simply would not occur. This mechanism was called theory of membrane double break. Studies held afterwards brought more subsidies to this theory, histologically proving the involvement of the Reissner membrane^9^^,^^16^.

We effectively created a severe lesion on the round window membrane of the guinea pigs of the present study. Although we did not aspirate perilymph through the opening created, we caused enough damage to bring about a major and sudden variation in intracochlear pressure, to the point of developing a secondary lesion to the membranous labyrinth as stated by Simmons. Thus, it is to be expected that besides the perilymph pressure changes and eventual loss of liquid through the fistula, the mixture of cochlear fluids could cause malfunctioning of the active mechanism of hair cell contractions, large on the basilar membrane, with SP amplitude reduction between PRE and PFT as seen, because of a lower degree of deflection of this membrane in response to sound. We also noticed a reduction between PFT and LPF, still in a lesser degree in both groups (OF and CF) and of a similar mode. How can we explain such behavior when there was no loss of fluids in the CF during the time period considered? Based on the natural trend of recovery and healing of the round and oval window membranes of guinea pigs^1^^,^^2^^,^^9^^,^^10^^,^^14^, we may conclude that they healed, even in the OF group, with partial recovery of functional units adjacent to the main lesion point, however insufficient to satisfactorily increase SP amplitude, justifying the behavior similarity between the groups. Or, as proposed by Simmons, there was a round window membrane closure, but the Reissner membrane lesion and the endo and perilymph mixture remained, justifying a worsening in the parameters. Another conclusion we may reach is that the membranous labyrinth lesions is more important for the recovery than the simple loss of perilymph, as advocated by other authors^9^^,^^16^. The hair cell behavior, either functional or anatomical, causes a raise in SP latency, since the stimulus takes longer to be formed. In the literature we found very few studies assessing SP amplitude variations; however they are in agreement with ours^12^^,^^16^.

Similarly to what happened with the SP, the AP would also be reduced in amplitude and latency increase for the same sound stimulus intensity, as it happened in our experiment, since there was a reduction in the number of recruited afferent fibers to trigger and make up the potential. We must stress that the AP potential is directly associated with the SP latency. This reinforces the idea that the AP latency depends on the SP latency, justifying its increase in our experiment. The AP amplitude reduction was also observed in previous studies^7^^,^^9^^,^^10^^,^^12^^,^^14^^,^^16^.

Comparing the SP/AP ratio results with the literature, we see differences. Studies have shown an increase in this ratio^7^^,^^12^^,^^17^^,^^18^; however, they used a different method from ours, with punctiform lesions and lesser cochlear involvement. On the other hand, contrary to other parameters assessed, there was a significant increase in the PFT and LPF ratio. Supposing that there was a closure of the shunt created on the Reissner membrane after the time period hereby considered and that despite the degree of lesion on the round window membrane, it healed even in guinea pigs with fistulas kept open (OF). This situation would bring about a replacement of cochlear fluids, capable of recovering those cells under functional damage and reestablishing pressure in the perilymphatic compartments to the point of increasing the SP/AP ration in PFT. It also justifies two issues still open. The lower intensity of SP and AP amplitude and latency drop in the PFT, and the trend towards better results in the OF group which we can see in the graphs presented, even if not significant from the stand point of statistics. Upon round window perilymph loss cessation, there is a lower gradient of hydrostatic pressure and lower mixture of endo and perilymph, with a greater trend toward Reissner membrane lesion healing. The earlier the correction of the first defect, the faster the liquid separation and the greater the chance of hair cell recovery after functional damage. A long term study could show a different behavior, more evident between the groups.

This trend towards a relatively fast and spontaneous healing in studies with guinea pigs can lead to the false assumption that the LPF would not be amendable to treatment in human beings. Good results among clinical treatments with steroids in patients with sudden hearing loss increase even further this challenge,^8^ especially considering the likelihood of viral infection^19^. Nonetheless, we must bear in mind that there is a very clear relation between the time of diagnosis and prognosis of the auditory and vestibular symptoms^20^. The longer the interval, the greater the residual auditory loss. This is a clear indication that surgery, when necessary, should not be delayed much^2^. Despite being indicated early on in the past,^4^^,^^5^ there is a current trend of delaying surgery for a period that varies between 10 and 14 days, when there are greater possibilities of a spontaneous closure, and then submit the patient to a surgical procedure in order to improve symptoms^1^^,^^3^. The physician must then weigh risks and benefits, with the patient's participation, in order to decide on the type of treatment to be instated, since the results obtained from the current study are restricted to guinea pigs and can not be considered in human beings.

## CONCLUSION

The results obtained allow us to conclude that the perilymphatic fistulas produced from the round window injury in guinea pigs tend to evolve naturally with a worsening in electrocochleography parameters; however, its surgical closure did not show statistically different results in relation to the non-treated group during the time of the study.
